# Warthin-like papillary thyroid carcinoma: a case report and comprehensive review of the literature

**DOI:** 10.3389/fendo.2023.1210943

**Published:** 2023-07-11

**Authors:** Abdel Mouhaymen Missaoui, Fatma Hamza, Wafa Belabed, Manel Mellouli, Mohamed Maaloul, Slim Charfi, Issam Jardak, Tahya Sellami-Boudawara, Nabila Rekik, Mohamed Abid

**Affiliations:** ^1^ Department of Endocrinology and Diabetology, Hedi Chaker University Hospital, Sfax, Tunisia; ^2^ Faculty of Medicine of Sfax, University of Sfax, Sfax, Tunisia; ^3^ Department of Nuclear Medicine, Habib Bourguiba University Hospital, Sfax, Tunisia; ^4^ Department of Pathology and Research Laboratory LR18SP10, Habib Bourguiba University Hospital, Sfax, Tunisia

**Keywords:** papillary thyroid carcinoma, thyroid neoplasms, thyroid nodule, warthin-like papillary thyroid carcinoma, chronic lymphocytic thyroiditis, case report, review of the literature

## Abstract

**Background:**

Papillary Thyroid Carcinoma (PTC) is the most frequent endocrine malignancy with a variety of histological presentations. Warthin-like Papillary Thyroid Carcinoma (WLPTC) is an uncommon neoplasm that is recognized as a distinct subtype of PTC in the WHO classification of thyroid tumors. In this report, we present a novel case of WLPTC in a female patient and provide an in-depth review of the available literature on its clinical, pathological, and therapeutic characteristics.

**Case presentation:**

A 27-year-old female patient was referred for neck swelling. Ultrasound showed two suspicious thyroid nodules leading to a thyroidectomy. She was diagnosed with intermediate-risk bifocal foci of classic PTC and WLPTC, arising from a background of chronic lymphocytic thyroiditis (CLT). This pT1b(m) N1b M0 malignancy was treated with adjuvant isotopic ablation and suppressive thyroxine therapy. The 1-year outcomes were favorable.

**Literature review:**

It covered articles published from 1995 to 2022, by searching PubMed and Google Scholar using specific terms. Out of 148 articles reviewed by two authors, 25 relevant articles were selected, including 13 case reports and 12 case series. The study included 150 cases of WLPTC. Data related to clinical presentation, imaging, histological features, management, and outcomes, were extracted. The mean age of diagnosis was 39 years, with a female predominance. The most common clinical presentation was neck swelling. Thyroid autoimmunity was positive in 71.6% of patients. Lymph node metastases were present in 28% of cases, with no reported distant metastases. Overall, the outcomes were favorable.

**Conclusion:**

WLPTC shares similar clinical and radiological presentations as classic PTC. The hallmark histological features of WLPTC are papillae lined with oncocytic tumor cells with papillary nuclear changes and lymphoid stroma. WLPTC is almost constantly associated with CLT. The management of WLPTC aligns with that of classic PTC with comparable stage and risk category, often resulting in favorable outcomes.

## Highlights

▪ WLPTC is a rare subtype of PTC that shares similar demographic, clinical, and imaging characteristics, as other forms of well-differentiated thyroid cancer.
▪ The hallmark histopathological features of WLPTC include papillary growth patterns, oncocytic neoplastic cells with nuclear features of papillary thyroid carcinoma, and a densely-lymphoid stroma.
▪ WLPTC has a strong co-occurrence with CLT and could be associated with thyroid dysfunctions and autoimmune disorders.
▪ WLPTC’s management follows the standard approach for treating other PTC of similar stage and risk category and has an excellent prognosis.

## Introduction

Globally, the incidence of thyroid cancer has risen over the past three decades, with an age-standardized incidence of 10.1 and 3.1 per 100,000 women and men, respectively (1, 2). The American Cancer Society predicts 43,720 new thyroid cancer cases in 2023, with 31,180 in women and 12,540 in men (3).

Papillary Thyroid Carcinoma (PTC) is the most common primary thyroid cancer, accounting for over 80% of all thyroid gland malignancies (4). It exhibits diverse morphological variations that are distinguished based on their architectural composition, proliferation pattern, and cellular and stromal features. In 2022, the World Health Organization (WHO) published the fifth edition of the classification of thyroid neoplasms, which placed significant emphasis on the histological subtyping of PTC (5). This was prompted by the correlation between BRAF-driven PTC and poor patient outcomes compared to RAS-driven PTC, as well as the overrepresentation of BRAF^V600E^ mutations in certain PTC subtypes, formerly referred to as “variants” (6, 7). In addition to classic PTC, the WHO now recognizes up to eight subtypes of PTC, including the infiltrative follicular, tall cell, columnar cell, Hobnail, solid, diffuse sclerosing, Warthin-like, and oncocytic PTC. The new classification requires a thorough subtyping of papillary microcarcinomas in the same manner as larger PTC and discourages the designation of microcarcinomas as a subtype of PTC (5–7).

The Warthin-like Papillary Thyroid Carcinoma (WLPTC) is a rare entity characterized by the presence of papillary architecture with prominent lymphocytic stroma in fibrovascular cores, resembling the Warthin tumor of the salivary gland. The neoplastic cells that line the papillary folds exhibit typical nuclear changes of papillary carcinoma. To date, fewer than 200 cases of WLPTC have been reported in the English literature (8, 9). Formerly affiliated with the oncocytic variant, the WLPTC is henceforth recognized as a distinct subtype, thus meriting focused attention and concise documentation to address pathological diagnosis and medical management (5, 7, 10).

In the present study, we present a new case of WLPTC in a bifocal PTC with lymph node metastases in a female patient and provide a comprehensive review of the literature regarding its clinical and radiological presentation, pathological features, management, and outcomes.

## Case description

A 27-year-old female patient was referred to the ENT department by her family physician due to the presence of two swellings in the lower neck. The patient had no personal medical history but reported a family history of hypothyroidism.

### Pre-operative findings

Upon neck examination, the patient was noted to have two indolent, firm thyroid nodules that had arisen six months earlier. Subsequent neck ultrasonography revealed bilateral, suspicious TIRADS V thyroid nodules, in addition to multiple lymphadenopathy located at levels III, IV, and VI. Regrettably, fine-needle aspiration was not performed at the time of the initial presentation due to the unavailability of skilled radiologists. Therefore, a total thyroidectomy was conducted, accompanied by bilateral central and lateral lymph node dissections.

### Gross specimen analysis

The thyroidectomy specimen consisted of a right lobe measuring 4x2.5x1.5 cm, a left lobe measuring 3x2.5x0.8 cm, and an isthmus measuring 2x0.5 cm. Upon macroscopic examination, two distinct thyroid nodules were identified: a 0.8 cm left-sided firm nodule with ill-defined borders and a white appearance (designated as Nodule A) and a 1.7 cm right-sided nodule (designated as Nodule B).

### Histopathological examination

**Figure 1:**
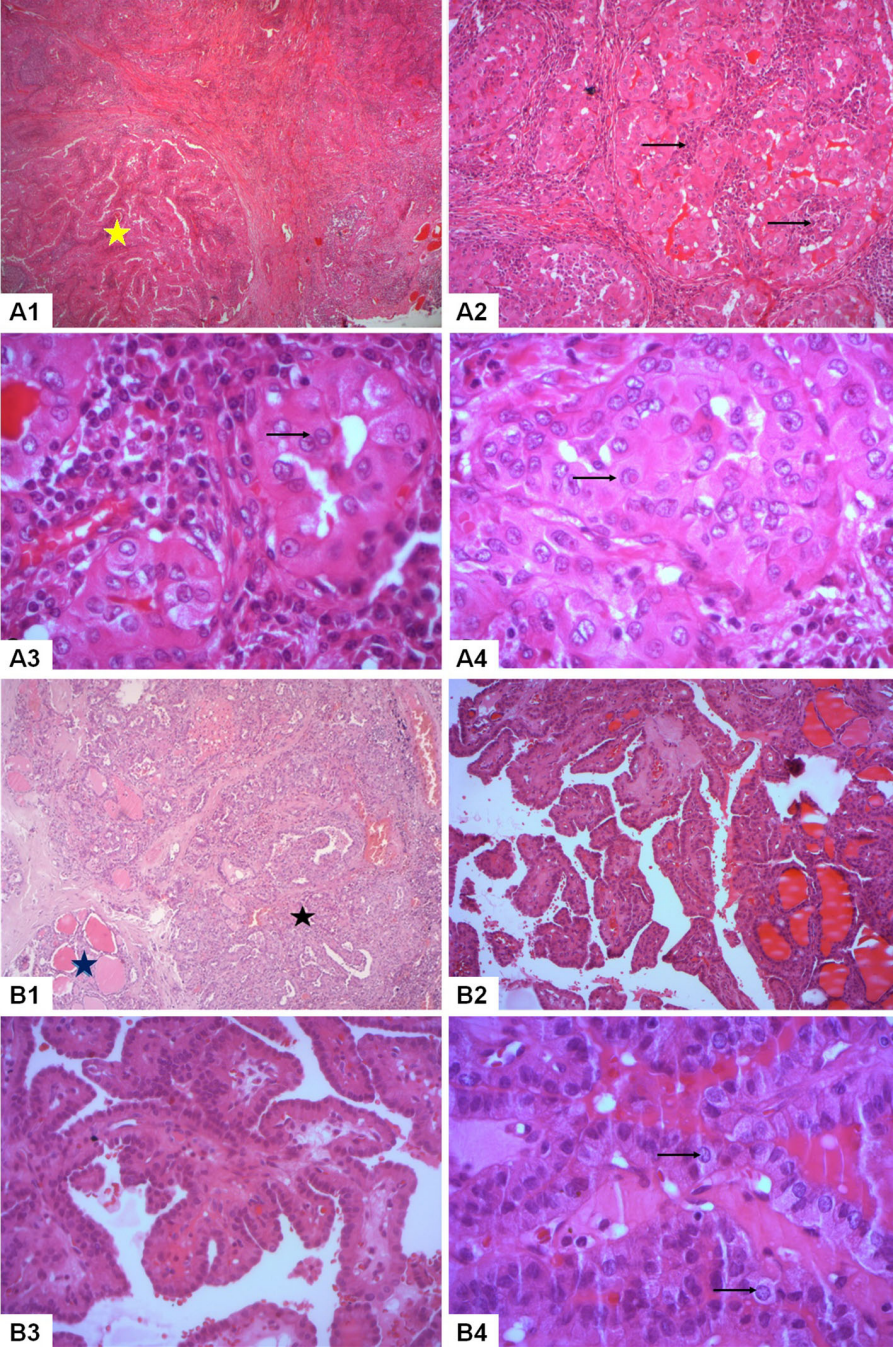
Histopathological analysis of a bifocal mixed presentation of papillary thyroid carcinoma with classic and warthin-like histology in our reported patient. Nodule A: Warthin-like papillary thyroid carcinoma (A1-A4). (A1) Histological examination revealed the presence of branching papillae composed of oncocytic tumor cells amidst a reactive lymphoid stroma (yellow star) (HEx50). **(A2)** A prominent lymphoplasmacytic infiltrate was identified within the cores of the papillae (→) (HEx100). **(A3)** The tumor cells exhibited nuclear enlargement, chromatin clearing, and nuclear groove (→) (HEx400). **(A4)** Pseudoinclusions were observed in the nuclei of the tumor cells (→) (HEx400). Nodule B: Classic papillary thyroid carcinoma (B1-B4). (B1) Classic papillary thyroid carcinoma (black star) was identified invading the thyroid parenchyma (blue star) (HEx50). **(B2)** The tumor featured a proliferation of complex papillae (HEx100). **(B3)** The cores of the papillae were identified as fibrovascular without a background of reactive lymphoid stroma (HEx200). **(B4)** The tumor cells exhibited distinctive papillary nuclear features such as nuclear enlargement and chromatin clearing (→) (HEx400).

Nodule (A) exhibited a papillary proliferation pattern with oncocytic features, characterized by abundant eosinophilic cytoplasm and large, irregular nuclei with papillary features. Moreover, it manifested extensive infiltration of lymphocytic cells, resembling the histological features of Warthin salivary tumors. Nodule (B) consisted of a papillary proliferation of epithelial tumor cells. We pointed out the presence of psammoma bodies and the infiltration into adjacent thyroid tissue (**Figure 1**). The underlying thyroid tissue was noted to have chronic lymphocytic thyroiditis lesions and lymphocytic follicles, and no evidence of vascular invasion was observed. Surgical margins were negative and examination of the dissected lymph nodes revealed multiple bilateral metastatic deposits of classic PTC.

**Figure 2:**
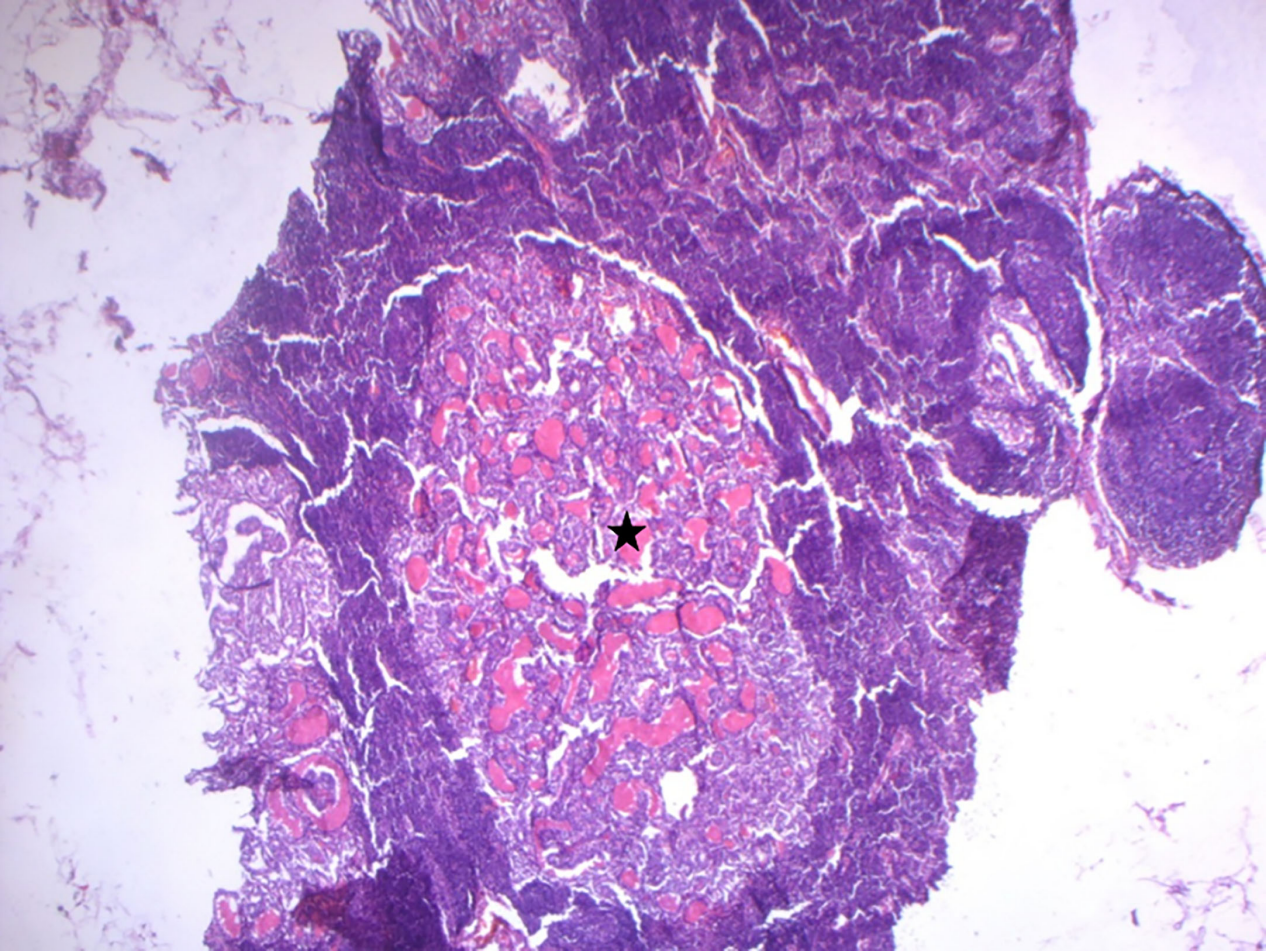
Microscopic findings of lymph node metastases. Histological examination of lymph node metastases revealed the typical histopathological features of conventional papillary thyroid carcinoma (indicated by the black star) (HE x100).

The patient was diagnosed with bifocal foci of PTC, characterized by a 0.8 cm Warthin-like subtype in the left lobe (Nodule A) and a 1.7 cm classic subtype in the right lobe (Nodule B), with bilateral lymph node metastases. The microscopic examination of these lymph nodes was consistent with the histopathological features of conventional PTC, as illustrated in **Figure 2**. The pathological staging, as per the AJCC 8th edition, was determined to be pT1b(m) N1b (11).

### Postoperative management and outcomes

Following surgery, TSH-stimulated serum Thyroglobulin was 0.8 ng/mL with positive anti-Thyroglobulin autoantibodies at 18.7 UI/mL (reference < 6.4). This ATA intermediate-risk bifocal PTC warranted adjuvant 131-radioiodine therapy (12). A Whole Body Scan after 100 mCi isotopic ablation showed intense iodine uptake in the thyroid bed. The patient was prescribed 175 µg daily suppressive thyroxine therapy. At the 1-year follow-up, results were favorable with undetectable Thyroglobulin, negative autoantibodies, and negative cervical ultrasound.

### Patient’s perspective

The patient and her family were satisfied with the management.

## Literature review

### Methodology

We conducted a comprehensive review of the literature on WLPTC, covering articles published between 1995 and 2022, by searching PubMed and Google Scholar databases using the search terms “Warthin-Like Papillary Thyroid Carcinoma” and “Oncocytic Variant Of Papillary Thyroid Carcinoma With Lymphocytic Stroma”. In total, 148 articles were retrieved and independently reviewed by two authors, AM and FH.

Articles reporting extra-thyroid Warthin tumors were excluded from our analysis as they fell outside the scope of our investigation. Duplicate articles were removed to avoid redundancy in the dataset. We excluded articles that lacked sufficient data supporting the diagnosis of WLPTC, based on a comprehensive evaluation of their title, abstract, and full-text content.

We only included cases that met the histopathological description of WLPTC as defined by the 2022 WHO classification of thyroid neoplasms (5, 7).

**Figure 3:**
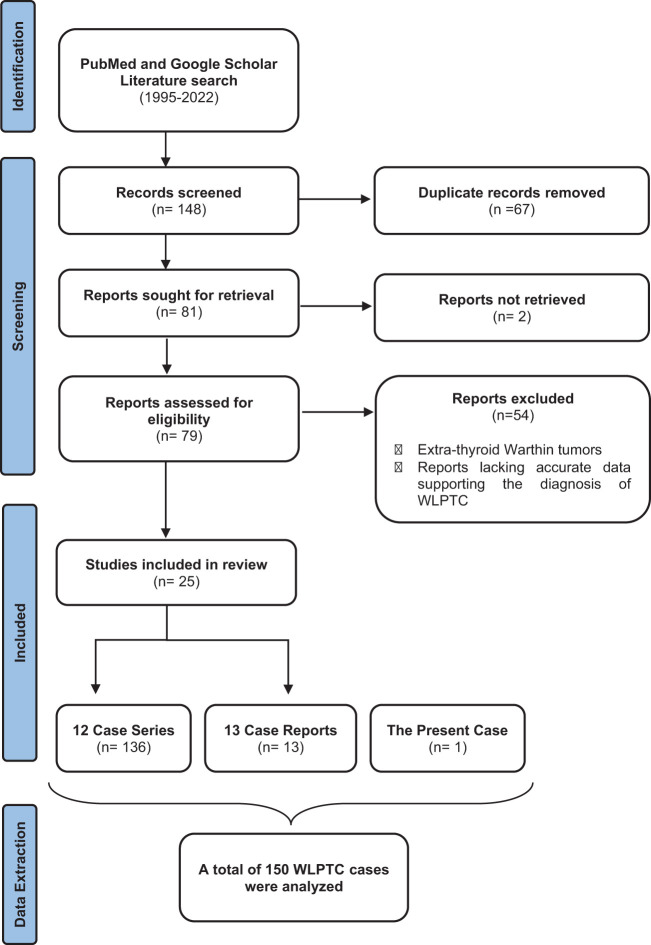
A graphical depiction of the data research and selection process in our comprehensive review of the literature. WLPTC, Warthin-Like Papillary Thyroid Carcinoma.

Following the aforementioned methodology, a total of 25 pertinent articles were ultimately selected for inclusion in our study. Among these, 13 were individual case reports, while the remaining 12 constituted case series (13–36). For each included study, a thorough manual extraction of data was conducted, focusing on various key aspects such as clinical presentation, imaging findings, histological features, management approaches, and outcomes. The methodology employed in our systematic review and the process of data selection are visually represented in **Figure 3**.

### Results

A total of 150 cases were considered (149 from previous literature and 1 from the present case report). The clinical presentation, radiological, pathological findings, management, and outcomes of WLPTC are summarized in **Table 1**.

**Table 1:** A comprehensive review of all 150 published cases of WLPTC.

Report	Number of cases	Sex	Mean age (yrs) [range]	Clinical presentation	Thyroid function	US findings	FNA findings	Tumor size (cm) [range]	Mutifocal DTC	CLT	Positive thyroid antibodies	Lymph node metastases	Distant metastases	Mean followup (months) [range]	Outcomes
Apel et al. (1995) (13)	13	1M : 12F	44 [26-66]	N/D	N/D	N/D	N/D	1.6 [0.3-3.5]	1/13	10/13	N/D	3/13	No	N/D	N/D
Tazawa et al. (1999) (14)	4	4F	50 [49-53]	N/D	N/D	N/D	N/D	1.9 [1.5-3]	0/4	4/4	N/D	4/4	No	N/D	N/D
Baloch et al. (2000) (15)	17	2M : 15F	[23-63]	16 NS 1 incidental	N/D	N/D	4 PTC features 2 CLT features 1 Atypical cells	1.3 [0.3-3.5]	N/D	17/17	N/D	3/17	No	N/D	12% Favorable 88% Unknown
D'antonio et al. (2000) (16)	3	1M : 2F	50 [43-56]	N/D	N/D	N/D	2 PTC features 1 NC	1.4 [1.3-1.5]	0/3	3/3	N/D	0/3	No	16 [3-3 6]	Favorable
Ludvíková et al. (2001) (17)	12	1M : 11F	64 [45-85]	NS	N/D	N/D	N/D	2.7 [1-5]	N/D	11/12	N/D	2/12	No	23 [4-51]	92% Favorable 8% Unknown
Kim et al. (2006) (18)	5	5 F	52 [33-65]	N/D	N/D	N/D	N/D	1.5 [0.9-2]	1/5	4/5	N/D	0/5	No	N/D	N/D
Paker et al.(2011) (19)	1	F	47	NS	N/D	Partially cystic hyperechogenic nodule	PTC features	3	0/1	1/1	N/D	0/1	No	11	Favorable
Paliogiannis et al. (2012) (20)	1	F	22	Incidental	HypoT	Hypoechoic nodule with irregular margins and rich vascularization.	PTC/ CLT	1.8	0/1	1/1	1/1	0/1	No	12	Favorable
Ersen et al. (2013) (21)	4	1M : 3F	39 (20–56)	N/D	N/D	N/D	2 SFM 1 PTC features 1 N/P	1.7 [0.9-2]	3/4	4/4	N/D	0/4	No	25 [22-26]	Favorable
Han et al. (2015) (22)	1	F	65	T1DM	EuT	Hypoechoic nodule with a hyperechoic spot, irregular margins, and rich vascularization	SFM	0.5	0/1	0/1	0/1	0/1	No	N/D	N/D
Padma et al. (2015) (23)	1	F	21	NS	HyperT	N/D	PTC/CLT	1.5	0/1	1/1	1/1	0/1	No	N/D	N/D
González-Colunga et al. (2015) (24)	1	F	36	NS	SCh	Hypoechoic nodule with irregular margins, central vascularity, incomplete halo, and microcalcifications	Atypical cells	1.7	0/1	1/1	1/1	1/1	No	12	Favorable
Kim et al. (2016) (25)	9	1M : 8F	50 [32-75]	N/D	1 Sch 8 EuT	5/9 Solid 7/8 Hypoechoic 1/8 Isoechoeic 4/8 Well-defined boarders 3/8 Irregular borders 3/8 Microcalcifications 9/9 Wider-than-larger	6 PTC features 1 SFM 1 N/P	1.1 [0.5-1.5]	2/9	9/9	N/D	3/9	No	72 [12-132]	Favorable
Sánchez Fuentes et al. (2017) [38]	1	F	33	Multinodular goiter	SCh	Hypoechoeic heterogeous goiter	N/D	0.3	0/1	1/1	1/1	0/1	No	12	Favorable
Vallonthaiel et al. (2017) (26)	1	F	30	NS	HypoT	Echogenic nodule	PTC/CLT	2	0/1	1/1	N/D	0/1	No	14	Favorable
Wajahat et al. (2018) (27)	1	M	42	NS	SCh	Hypoechoic nodule with regular margins	PTC features	3.5	0/1	1/1	N/D	0/1	No	N/D	N/D
Hirokawa et al. (2018) (28)	17	4M : 13F	52.2 [31–77]	N/D	1 TypoT 16 EuT	N/D	N/D	1.6 [0.6–3.2]	0/17	14/17	14/17	8/17	No	N/D	N/D
Xavier-Júnior et al. (2019) (29)	3	3F	57 [54-60]	NS	N/D	2 Hypoechoeic nodules 1 Solid nodule with cystic changes	2 Atypical cells 1 SFM	1.9 [1-2.5]	1/3	3/3	N/D	1/3	No	N/D	N/D
Ning et al. (2019) (30)	32	5M : 27F	51 [30-74]	3/32 NS 29/32 Incidental	14/32 HyperT 2/32 SCH 1/32 SCh 15/32 EuT	32/32 solid 26/33 Hypoechoic 6/33 Taller-than-wider shape	9 /31 PTC features 22/32 SFM 1/32 N/P	1.2 [0.5-3]	2/32	28/32	22/32	8/32	No	N/D	N/D
Villalba Ferrer et al. (2020) (31)	1	F	59	NS	EuT	Solid hypoechoic nodule with no halo, scarce vascularization and ill-defined margins	Benign cytology	2	0/1	1/1	1/1	0/1	No	18 months	Favorable
Olmos et al. (2020) (32)	17	1M : 16F	39	N/D	N/D	N/D	N/D	1.5 [0.4-3]	10/17	10/17	11/17	7/17	No	2 (0.6-6.6)	23% Favorable 77% Indeterminate
Agrawal et al. (2021) (33)	1	F	43	NS	EuT	Solid-cystic, wider-than-taller hypoechoic nodule with a few echogenic foci	PTC/CLT	2.5	1/1	1/1	N/D	1/1	No	N/D	N/D
Curto et al. (2021) (34)	1	F	43	Multiple sclerosis	EuT	Nodule with hyperechogenic spots	N/D	0.8	0/1	0/1	0/1	0/1	No	7	Favorable
Sanyal et al. (2022) (35)	1	F	60	NS	EuT	Hypoechoic nodule with complete peripheral halo and ill-defined margins	PTC features	4.5	0/1	1/1	N/D	0/1	No	6	Favorable
Hu et al. (2022) (36)	1	F	69	Multinodular goiter	N/D	Solid hypoechoic wider-than-larger nodules	N/D	1.3	1/1	1/1	N/D	0/1	No	N/D	N/D
Present case	1	F	27	2 NS	EuT	Hypoechoic taller-than-wider nodules	N/P	0.8	1/1	1/1	1/1	1/1	No	12	Favorable
Summary	150	132 F (88%) 18 M (12%)	39 [20-77]	72 Unknown 43 NS 31 Incidental 2 Goiter 2 AID	45 EuT 3HypoT 5 SCh 2 SCH 15 HyperT 80 N/D	Variable	39% PTC features 42,2% SFM 3,1% CLT 6,3% PTC/LT 6,3% Atypical cells 3,1% benign or NC	2.1 [0.3-5]	81% Unifocal 19% Multifocal	69.3%	71.6%	28%	No	Variable ranging from 6 months to 11 years	Commonly favorable

PTC, Papillary Thyroid Cancer; WLPTC, Warthin-like Papillary Thyroid Cancer; N/D, Not Detailed; N/P, Not Performed; M, Male F, Female; NS, Neck Swelling; EuT, Euthyroid; HypoT, Hypothyroidism; SCh, Subclinical Hypothyroidism; HyperT, Hyperthyroidism; SCH, Subclinical Hyperthyroidism; SFM, Suspicious for Malignancy; CLT, Chronic Lymphocytic Thyroiditis; PTC/CLT, Papillary Thyroid Cancer on a Background of Chronic Lymphocytic Thyroiditis; NC, Non-Conclusive; DTC, Differentiated Thyroid Cancer.

The mean age of patients at the time of diagnosis was 39 years (range: 20-77 years) with a female predominance: 132 females (88%) compared to 18 males (12%). The initial clinical presentation of WLPTC was not specified in 72 cases. In the remaining 78 cases, the most common reason for consultation was neck swelling (43/78, 55.1%). In two cases, the subtype arose in a background of multinodular goiter. WLPTC was incidentally diagnosed in 31/78 cases (39.7%) on imaging performed for other reasons.

Thyroid function was assessed in 70 of the 150 reported cases, with the majority being euthyroid (45/70, 64.3%). There was a significant rate of thyroid function disorders associated with WLPTC (25/70, 35.7%), including overt hypothyroidism (n=3), subclinical hypothyroidism (n=5), overt hyperthyroidism (n=15), subclinical hyperthyroidism (n=2). Thyroid autoimmunity was found in 71.6% of the tested patients.

The ultrasonographic characteristics of thyroid nodules harboring WLPTC were heterogeneous. FNA was performed in some cases and often concluded with PTC cytological features (39%) or suspicious for PTC (42.2%).

The mean tumor size of WLPTC was 2.1 cm (range: 0.3-5 cm) with multifocal disease reported in 19% of patients. Chronic lymphocytic thyroiditis was documented in 69.3% of the cases. Lymph node metastases were present in 28% of cases. Most of the cases reported do not provide precise information about the documented intralymphatic spread of the tumor, including intralymphatic psammoma bodies or viable tumor emboli. Distant metastases have never been reported. Postoperative management was rarely detailed in the reported cases. The mean follow-up of patients treated for WLPTC was variable, ranging from 6 months to 11 years. The outcomes were most commonly favorable. One single author reported an indeterminate response in 13 patients (32). No cases of recurrent WLPTC have been reported so far.

## Discussion

The escalation in the number of diagnosed PTCs has induced a transition in the epidemiology of PTC subtypes, wherein certain subtypes, hitherto deemed uncommon, have now emerged as more prevalent. The description of WLPTC was first put forth in 1995 by Apel et al. and referred to as “Oncocytic variant of PTC with Lymphoid Stroma” (13). Subsequently, additional cases were reported under the same nomenclature. Since 2017, the WHO officially classify WLPTC as a distinct subtype of PTC (5–7).

### Epidemiology, demographic, and clinical presentation

Thus far, the precise prevalence of WLPTC is unknown, likely due to its rarity and frequent misdiagnosis as an oncocytic variant by many pathologists. Jun et al. estimated its prevalence to be 0.2% of all PTCs based on the examination of 8,179 PTC specimens at a reference Korean thyroid cancer center (37). This proportion is expected to increase with the implementation of the updated pathological classification.

The demographic and clinical features of WLPTC are similar to those of other differentiated thyroid cancers. WLPTC mainly affects young individuals aged 30 to 50 and is less common in older patients. In the 150 reviewed cases, we observed a marked female predominance of 88%, consistent with the general epidemiology of all PTCs.

The initial symptom is often a painless cervical swelling, which was present in about half of the cases (15, 20, 30). The availability of imaging techniques has led to increased incidental diagnoses of WLPTC. Rarely, WLPTC may present as a multinodular goiter, as reported in only two cases (36, 38).

### Ultrasonographic and fine-needle aspiration findings

The literature on ultrasonographic data for WLPTC is inconsistent. Ning et al. conducted a study of 32 cases and found that all cases scored higher than 5 points on the ACR TI-RADS scale. 7 cases were classified as TR4 and 26 cases as TR5. Almost all nodules were solid or nearly solid (97%) and highly hypoechoic (78.8%). They were also wider in shape than tall (81.8%) (30). These findings agree with those reported by Kim et al., who encountered that the majority of WLPTC nodules were solid (62.5%), hypoechoic (75%), and wider in shape than tall (100%) (25). We underline that pathological examination is crucial for a definitive diagnosis of WLPTC, as ultrasonic findings alone are not specific enough (20).

FNA of WLPTC-affected thyroid nodules typically displays an overlapping pattern of papillary nuclear features, oncocytic cells, and chronic lymphocytic thyroiditis (CLT) (9, 20, 24, 26). However, the interpretation of FNA results by pathologists can be challenging and often results in inconclusive findings. Our systematic review found that only 6.3% of FNA examinations showed typical cytological features of WLPTC. Most specimens were diagnosed as PTC or suspicious for PTC. In some cases, FNA was misdiagnosed as benign (15, 16, 21, 25, 29–31). This underscores the importance of considering definitive histological examination for accurate diagnosis of WLPTC.

### Histopathological diagnosis

Of note, the two hallmark histological features of conventional PTC are the presence of papillae and nuclear changes (10, 39). The papillae consist of a central fibrovascular stalk covered in a neoplastic epithelium and exhibit a predominant papillary pattern throughout the tumor. Nuclear abnormalities in PTC cells can include variations in size and shape, contour irregularities, chromatin changes, intranuclear grooves, and pseudoinclusions. These cells typically have a slightly eosinophilic cytoplasm with rare or absent mitotic activity. Classic PTC often includes psammoma bodies and abundant fibrous stroma (10, 39, 40).

As for WLPTC, it often replicates similar papillary architecture but with a dense lymphoplasmacytic infiltrate within the core of the papillae. These papillae are typically lined with tumor cells that display oncocytic changes. They feature an abundant, granular, eosinophilic, mitochondria-rich cytoplasm while preserving the typical papillary nuclear characteristics. This variant usually develops in the background of CLT (5, 39, 40).

### Differential diagnoses

In some instances, WLPTC can be misdiagnosed as tall cell or oncocytic subtypes of PTC (9, 17, 41). In **Table 2**, we compare the distinctive histological characteristics of WLPTC to these differential diagnoses. Likewise, differentiating between CLT and WLPTC can be challenging on some specimens. In fact, CLT also presents with widespread lymphoplasmacytic infiltration and oncocytic changes recognizable in the follicular epithelial cells. In such situations, the lack of the characteristic papillary nuclear changes in CLT is a key distinguishing factor (42).

**Table 2:** Distinctive characteristics of WLPTC compared to other PTC subtypes.

Histological features	Classic PTC	WLPTC	Oncocytic PTC	Tall cell PTC
**Growth pattern**	Papillary	Papillary	Papillary or follicular	Papillary
**Cell apperance**	Slighly eosinophilic cytoplasm	Oncocytic changes	Oncocytic changes	Oncocytic changes ; three times as tall as wide
**Papillary nuclear features**	Yes	Yes	Yes	Mainly elongated dark nuclei
**Lymphocytic infiltrate**	Slight	Dense	Absent	Slight
**Underlying CLT**	Possible	Very common	Absent	Rare
**Extrathyroid extension**	Possible	Rare	Possible	Common
**Vasular invasion**	Rare	Rare	Possible	Common
**LN metastases**	Common	Common	Common	Very common
**Distant metastases**	Possible	Rare	Possible	Common

PTC, Papillary Thyroid Cancer; WLPTC, Warthin-like Papillary Thyroid Cancer; CLT, Chronic Lymphocytic Thyroiditis; LN, Lymph Node.

### Aggressive features in WLPTC

WLPTC, similar to classic PTC, can present as a single lesion or in association with multifocal PTC disease, which occurs in 19% of all reported cases of WLPTC, as indicated by our review (21). Like all PTCs, invasion of lymph vessels is a common feature and occurs in 28% of patients with WLPTC. This rate is yet lower than in classic PTC, where up to 50–60% of patients already have lymphatic spread at the time of diagnosis (43). In the current case, our patient had multiple metastatic deposits at different lymphatic levels. This high lymphogenic metastatic potential was not attributed to the WLPTC foci per se but rather resulted from the synchronous classic PTC lesions coexisting within the patient’s thyroid. This assertion is substantiated by the histopathological examination of lymph nodes conducted in our reported case.To date, no cases of distant metastasis have been reported in WLPTC patients, compared to classic PTC where less than 10% of patients experience distant spread (12, 44).

### Association between WLPTC and thyroid disorders

WLPTC has a strong co-occurrence with CLT, with nearly 70% of histological findings in our review demonstrating this association. This rate is significantly higher compared to the typical coexistence between PTC and CLT, which is estimated to range from 0.5% to 38% (45). The exact relationship between PTC and CLT remains a subject of debate. Some authors suggest that CLT may trigger carcinogenesis through the expression of inflammatory signals and genomic instability, thereby affecting the biological behavior of PTC (46). Others hypothesize that CLT represents a histological manifestation of a humoral and cytotoxic T cell-mediated immune response against PTC that acts as a protective mechanism controlling tumor growth (45, 47). Given the favorable outcomes recorded in the 150 cases of WLPTC and its lack of aggressive characteristics, our inclinations lean towards endorsing the latter hypothesis.

The presence of CLT alongside WLPTC may either be symptom-free or manifest in the form of various thyroid dysfunctions. The majority of individuals diagnosed with WLPTC are euthyroid. Out of the 70 patients who underwent thyroid function testing in our review, 35.7% were found to have thyroid dysfunction, with 8 displaying hypothyroidism and 17 exhibiting hyperthyroidism. A significant proportion, 71.6%, of patients tested positive for thyroid autoimmunity. Some cases also reported the co-existence of WLPTC with other autoimmune conditions such as type 1 diabetes mellitus and multiple sclerosis (22, 34).

### Biomolecular findings

The presence of the BRAF^V600E^ mutation is a frequent occurrence in PTC, with a prevalence ranging from 36% to 69%. This mutation has been associated with a less favorable clinicopathological and metastatic behavior of PTC (48, 49). Xing et al. demonstrated a significant correlation between the presence of the BRAF^V600E^ mutation and the recurrence of PTC in a multicenter study (50). Our patient was not genetically sequenced as the procedure is not part of the routine protocol at our institution. According to Trovisco et al., who sequenced 134 consecutive cases of PTC, the BRAF^V600E^ mutation was found in 75% of WLPTC cases compared to 53% in conventional PTCs (51). The high prevalence of the BRAFV600E mutation in WLPTC contrasts with the generally excellent prognosis of this subtype. Further investigations on larger sample sizes are required to fully clarify the role of this genetic alteration in WLPTC.

### Management and follow-up

The management of WLPTC follows the standard approach for treating other classic PTC of similar stage and risk category and typically involves total or partial thyroidectomy (12, 52). As the diagnosis of WLPTC is commonly confirmed through surgical specimens, there is limited scope for preoperative measures. In high-risk patients, additional procedures such as completion thyroidectomy, lymph node dissection, and radioiodine ablation may be required. In the reported cases, we lack information regarding the specifics of postoperative management, such as ATA risk classification, radioactive iodine treatment, and TSH suppression therapy for WLPTC. As for the case of our patient, she was diagnosed with an intermediate-risk WLPTC and underwent total thyroidectomy, lymph node dissection, and received a 100 mCi isotopic ablation and TSH suppression therapy.

The follow-up of patients treated for WLPTC is variable in published studies, and the precise modalities are not well defined. However, all authors agree on the favorable prognosis of this subtype. Overall and disease-free survival rates of patients with WLPTC are comparable to those with conventional PTC. A study by Olmos et al. confirmed that both biochemical and structural recurrence rates are similar between WLPTC and classic PTC. Regarding treatment response, WLPTC had a lower rate of excellent response compared to conventional PTC (32). This is due to a high rate of positive thyroglobulin autoantibodies in WLPTC, which impairs the achievement of an excellent response (12). To clarify the follow-up process for WLPTC, we suggest utilizing both thyroglobulin and thyroglobulin autoantibodies titer together as monitoring tools, as was done in our patient’s case.

## Conclusion

To the best of our knowledge, this is the largest comprehensive review of the literature that includes all previously published cases of WLPTC. In our work, we emphasized the typical clinical and pathological description of WLPTC and its association with thyroid function and autoimmune disorders. This can assist clinicians and pathologists in providing an accurate diagnosis and personalized healthcare for patients with WLPTC. Further studies are warranted to better understand the genetic basis and carcinogenic mechanisms of WLPTC and to refine its surgical and postoperative management strategies.

## Data availability statement

The original contributions presented in the study are included in the article. Further inquiries can be directed to the corresponding author.

## Ethics statement

This study which involves human participant was approved by Ethics Committee of Sfax University Hospitals. The participant provided the written informed consent to participate in this study.

## Author contributions

AM, FH, MoM, and IJ conceived the case study and the review methodology. AM and FH carried out the review of the literature. AM, FH, and WB drafted the manuscript. MaM and SC performed the histopathological analyses and designed the figures. TS-B, NR, and MA supervised the research progression and approved the final version of the manuscript. All authors contributed to the article and approved the submitted version.
